# Epigenome Engineering in Cancer: Fairytale or a Realistic Path to the Clinic?

**DOI:** 10.3389/fonc.2015.00022

**Published:** 2015-02-06

**Authors:** Fahimeh Falahi, Agustin Sgro, Pilar Blancafort

**Affiliations:** ^1^Cancer Epigenetics Group, Harry Perkins Institute of Medical Research, School of Anatomy, Physiology and Human Biology, The University of Western Australia, Perth, WA, Australia

**Keywords:** epigenetics, epigenome editing, genome editing, histone modifications, DNA methylation, zinc finger proteins, TALEs, CRISPR/dCas9

## Abstract

Epigenetic modifications such as histone post-transcriptional modifications, DNA methylation, and non-protein-coding RNAs organize the DNA in the nucleus of eukaryotic cells and are critical for the spatio-temporal regulation of gene expression. These epigenetic modifications are reversible and precisely regulated by epigenetic enzymes. In addition to genetic mutations, epigenetic modifications are highly disrupted in cancer relative to normal tissues. Many epigenetic alterations (epi-mutations) are associated with aberrations in the expression and/or activity of epigenetic enzymes. Thus, epigenetic regulators have emerged as prime targets for cancer therapy. Currently, several inhibitors of epigenetic enzymes (epi-drugs) have been approved for use in the clinic to treat cancer patients with hematological malignancies. However, one potential disadvantage of epi-drugs is their lack of locus-selective specificity, which may result in the over-expression of undesirable parts of the genome. The emerging and rapidly growing field of epigenome engineering has opened new grounds for improving epigenetic therapy in view of reducing the genome-wide “off-target” effects of the treatment. In the current review, we will first describe the language of epigenetic modifications and their involvement in cancer. Next, we will overview the current strategies for engineering of artificial DNA-binding domains in order to manipulate and ultimately normalize the aberrant landscape of the cancer epigenome (epigenome engineering). Lastly, the potential clinical applications of these emerging genome-engineering approaches will be discussed.

## Introduction

Epigenetic mechanisms including histone modifications, DNA methylation, and non-coding RNAs (ncRNAs) are essential for the mitotic maintenance of gene expression. Indeed, aberrant epigenetic regulation is associated with several pathological processes such as cancer. While for many decades much focus has been placed on genetic mutations as primary cause of cancer and cancer progression, the discovery of reversible epigenetic alterations in cancer has illuminated novel and exciting therapeutic avenues (1).

One primary function of the epigenetic processes is purely structural: packaging the genetic information in the nucleus of eukaryotic cells. The human genome contains approximately 3 billion base pairs (bps) of DNA, which are organized in 23 chromosomes. Each diploid cell with 46 chromosomes contains 6 billion bps of DNA. As each base measures 0.34 nm, approximately 2 m of DNA must be condensed in the nucleus of each diploid cell. Histone proteins are key players responsible for organizing the long fibers of DNA within the nucleus and the complex of DNA with histones is referred as chromatin.

Histone proteins H1, H2A, H2B, H3, and H4 are small and positively charged molecules involved in DNA compaction. Approximately 147 bps of superhelical DNA is wrapped around dimers of histones H2A, H2B, H3, and H4 composing the nucleosome core particle (2). The protruding N-terminal tails of histones undergo post-translational chemical modifications including but not limited to acetylation (ac), methylation (me), phosphorylation (P), ubiquitination (ubi), and SUMOylation (SUMO) (3, 4). According to the nomenclature describing histone modifications, first the histone is named, followed by the modified amino acid residue along with its position in the protein and lastly the type of chemical modification is included (for instance, H3K9me3 designates 3 methylation groups on lysine 9 in the histone H3 tail) (5). Arginine (R) and/or lysine (K) residues in H3 are commonly found acetylated or methylated (4).

Histone post-transcriptional modifications are of reversible nature and are incorporated or removed by a broad range of epigenetic modifier enzymes (epi-enzymes) (6). These chemical modifications (“marks”) confer very important biochemical roles to their histone substrates (7). Notably, histone proteins with different covalent modifications can be associated with either repressive or active forms of chromatin and thus they control gene expression status (4, 8). In general, H3K9me2/3 and H3K27me3 are accompanied with gene repression, whereas H3K4me3 and H3/H4 acetylation are often associated with active gene expression (9).

In addition to histone post-transcriptional modifications, chromatin structure and function is regulated by DNA methylation. Indeed, DNA methylation was the first identified epigenetic mark (10). DNA methylation is catalyzed by DNA methyltransferase enzymes (DNMTs) and mainly occurs at the 5′-carbon of a cytosine base that is generally preceding guanine (CpG). This mark is often referred to as the fifth DNA base (in addition to A, T, C, and G), and it plays a pivotal role in gene expression regulation, for example, by preventing transcription factors to bind and/or by recruiting repressive protein complexes to the DNA (11). Although DNA methylation is relatively more stable than the histone post-transcriptional modifications, it is also of reversible nature and can be removed by either passive (e.g., during DNA replication) or active processes [e.g., catalytically removed by specific enzymes including 10–11 translocation (TET), thymidine DNA glycosylase (TDG), or by activation-induced deaminase (AID) families (12–14)]. Both active and passive DNA demethylation mechanisms are crucial for normal development and cellular differentiation in mammalians (13, 15).

Non-coding RNAs (ncRNAs) have emerged as important epigenetic regulators in crucial biological processes such as differentiation and development (16). ncRNAs comprise several types of short ncRNAs, including microRNAs (miRNAs), short interfering RNAs (siRNAs), and PIWI-interacting RNAs (piRNAs). In addition to small ncRNAs, long ncRNAs (lncRNAs) of 200 nucleotides or more in length, are also implicated in chromatin organization and in the control of gene expression (17). For example, the lncRNA *HOTAIR* (*HOX antisense intergenic RNA*) regulates the *HoxD* loci and is found overexpressed in primary breast tumors and metastases. Thus, the level of *HOTAIR* expression represents a useful biomarker to predict metastatic disease. Importantly, *HOTAIR* expression is associated with changes in histone post-transcriptional modifications that are mediated by recruitment of histone modifier enzymes such as the polycomb repressive complex (PRC2), which is a histone H3 lysine 27 (H3K27) methylase (18).

In addition to histone modifiers, ncRNAs have been reported to mediate the recruitment of DNA methyltransferases, promoting *de novo* DNA methylation and transcriptional silencing. A recent report has demonstrated that ectopic expression of a promoter associated non-coding RNA (pRNA) induced DNA methylation, heterochromatin formation, and silencing of a ribosomal RNA gene promoter by recruitment of DNMT3b. Along with an increased DNA methylation, an induction of inactive histone modifications, and a concomitant decrease of active histone modifications were observed (19).

Another class of ncRNAs regulating DNA and histone methylation is referred as “piRNAs,” which interact with Piwi-containing proteins. piRNAs regulate the expression of amplified genomic sequences such as transposons (e.g., LINE-1 elements) preventing their expression in the germinal line (20, 21). Proteins belonging to the argonaute (Ago) family are small RNA and DNA-guided endonucleases involved in host-defense mechanisms that are highly conserved across kingdoms, from archaea to eukaryotes. In the cytoplasm, Ago proteins are critical for processing of miRNAs and for post-transcriptional gene silencing in complex with RNA induced silencing complex, RISC. In addition, an emerging function of nuclear Ago proteins involves the epigenetic control of gene expression via the recruitment of chromatin modifiers (e.g., H3K9 and H3K4 methytransferases), resulting in either repression or activation of gene expression (22).

In the following sections, we will overview the role of epigenetic modifications (DNA methylation and histone modifications) in cancer and describe novel technologies for genome engineering in cancer cells.

## Epigenetics in Cancer

Genome-wide maps of epigenetic modifications in normal and cancerous cells have provided insights into the involvement of specific epigenetic processes in cancer initiation and progression. Epigenetic enzymes are frequently deregulated in cancer relative to the normal tissue and the resulting epigenetic patterns associated with abnormally altered expression of genes involved in various cellular pathways including cell proliferation, cell differentiation, and DNA repair (23).

The altered status of histone methylation and acetylation, the global loss or low levels of acetylation of histones H3 and H4 (H3K9ac, H3K18ac, and H4K12ac) and methylation of histones H3 and H4 (H3K4me2 and H4K20me3) are among the most outstanding features in cancer (24, 25). In addition, the cell-specific pattern of DNA methylation is severely disrupted in cancer. It has been determined that the cancer genome is globally *hypo*methylated, which is associated with chromatin instability. In contrast, the promoters of several silenced tumor suppressor genes in cancer are *hype*rmethylated, which is consistent with the role of DNA methylation in gene silencing in these promoter contexts (2, 26, 27). The tight correlation between the disrupted epigenome and deregulated gene expression in cancer suggests that epigenome editing can be a potential novel approach for normalizing the gene expression profile of cancerous cells.

## Epigenetic Modifiers as Emerging Targets for Cancer Therapy

Epigenetic modifications are catalyzed and maintained by epigenetic modifier enzymes (epi-enzymes). In principle, the inhibition of specific epi-enzymes that are overactive in cancer cells can potentially reverse the incorporation of epigenetic mutations making epi-enzymes very attractive targets for cancer therapy.

There is a growing list of epigenetic drugs (epi-drugs) that have been developed for the specific inhibition of epi-enzymes. Epi-drugs comprise mainly DNA methyltransferase inhibitors (DNMTis) and histone deacetylase inhibitors (HDACis). However, new epi-drugs including histone methyltransferase inhibitors (HMTis) and a second-generation of DNMTis are being developed and tested for the mixed lineage rearranged leukemia (HMTis), advanced hepatocellular carcinoma, ovarian cancer, myelodysplastic syndrome, and acute myeloid leukemia (DNMTis) (28). Several DNMTis (e.g., azacitidine and decitabine) and HDACis (e.g., vorinostat and romidepsin) are FDA-approved and have been used in clinical trials for several years for treatment of hematological malignancies (29) and not until recently used for the treatment of solid tumors (23, 28, 30).

The efficacy of particular epi-drugs has highly improved in form of combinatorial treatments with other epi-drugs or with other forms of therapy such as hormonal therapy and chemotherapy. In these cases, the epi-drugs are anticipated to sensitize resistant types of cancer to their current therapies. For example, by demethylating and re-expressing estrogen receptors, specific epi-drugs are expected to sensitize resistant breast cancer cells to hormonal therapy (23). The molecular mechanisms of most epi-drugs are based on the inhibition of specific epi-enzymes that either remove acetyl groups from histones or add methylation groups (to histones or DNA), resulting in gene up-regulation. In this regard, tumor suppressor genes that are silenced by epigenetic mechanisms are anticipated to reactivate their expression upon epi-drug exposure. However, because of the lack of target selectivity, epi-drugs can cause genome-wide effects such as up-regulation of prometastatic genes (31, 32) and disruption of multiple cellular pathways due to unwanted effects of epi-drugs on some proteins such as P53, nuclear factor-κB, nuclear receptors, c-Myc, heat-shock protein-90, and so on. For example, acetylation of transcription factor P53 by histone acetyltransferase P300 can lead to activation of P53 and thus may result in a change in expression of the genes, which are regulated by P53 (33, 34). In order to improve the specificity and efficiency of epigenetic therapy, epigenetic reprograming in a gene-targeted manner (epigenome editing) represents an exciting alternative option.

## Genome-Editing Approaches

Gene therapy is becoming more and more attractive in cancer research as it might represent an alternative treatment for several difficult to treat cancers that suffer from resistance to the current therapies, including hormone therapy. Historically, however, the gene therapy field has experienced ups and downs due to the undesirable effects of the therapy, which even led to the death of patients and to the development of leukemia-like symptoms during the trials. However, since the early 2000s and by developing safer and more efficient gene delivery technologies, gene therapy has been successfully implemented for the treatment of patients with different diseases including metastatic melanoma (35).

By means of gene therapy, critical genes can be introduced in the genome or their expression regulated in order to inhibit cancer cell growth. In some classic gene therapy approaches, the target gene is either knocked-down using interfering ncRNAs, e.g., siRNAs or is expressed by introducing ectopic cDNAs into the cells. These approaches have their own limitations, for instance, the continual administration of ncRNAs or cDNA is necessary. In addition, one specific ncRNA or cDNA might not be sufficient for the repression or up-regulation of all possible various isoforms of a gene. Finally, the lack of an inefficient delivery system is the major obstacle that remains to be addressed. In order to solve the problem of the transient effect of ncRNA or cDNA, targeting a gene directly at the DNA level is a promising new emerging strategy. To target a given gene, DNA-binding domains must be developed with ideally single locus selectivity, and these domains are next utilized in several genome-editing approaches.

## DNA-Binding Domains and Their Specificity to Their Targets

**Figure 1:**
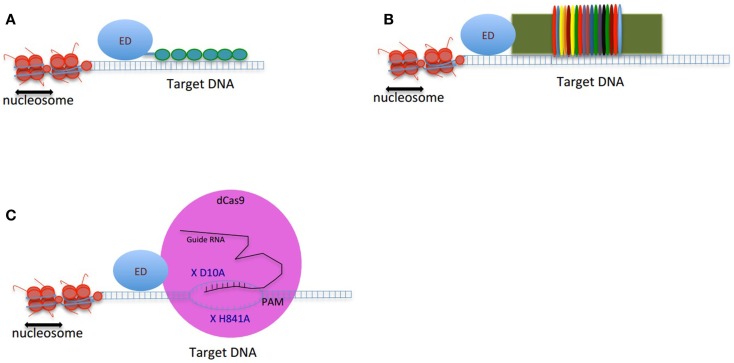
**Schematic figure of genome-editing tools composed of an effector domain (ED) fused to a DNA-binding domain: (A) ZFP, (B) TALEs, and (C) CRISPRs**. The double-strand DNA is shown as two parallel blue lines, the vertical small lines connecting the two strands of DNA are representing the hydrogen bonds between nucleotides. The small green circles in **(A)** are presenting 6-finger ZFP. The colorful thin ovals in **(B)** are representing tandem repeats of TALEs. In **(C)**, the pink large circle represents dCas9 protein. X D10A and X H840A are two mutations deactivating endonuclease activity of dCas9. The guide RNA is shown in black. The target binding site of guide RNA is located upstream of PAM. The small vertical lines between guide RNA and the target region of DNA are showing the hydrogen bonds.

Genome targeting tools are the essential components of the genome correction approaches. To date, several types of DNA-binding proteins have been developed to target specific loci in the genome. Zinc finger proteins (ZFPs), transcription activator-like effectors (TALEs), and clustered regularly interspaced short palindromic repeats (CRISPRs) are the most commonly exploited DNA-binding proteins, which are engineered to target a genomic sequence of interest (Figure 1).

**Figure 2:**
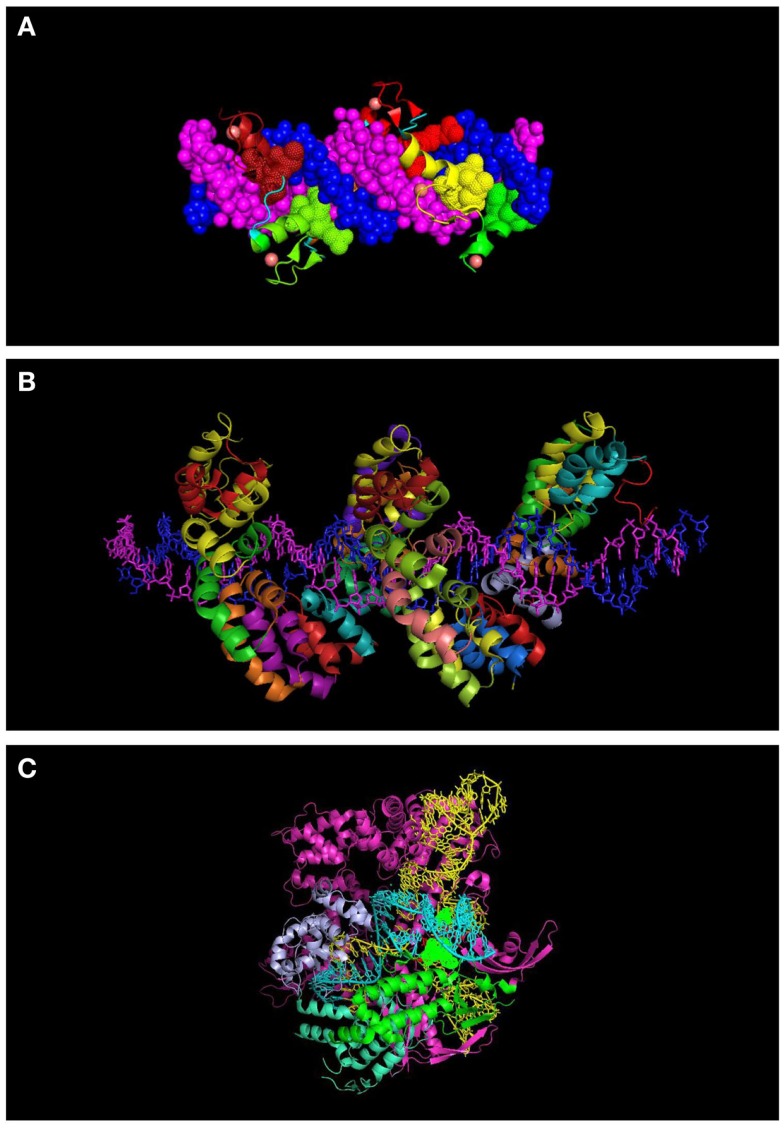
**Molecular representation of DNA-binding proteins used in genome engineering (A) 6-finger ZFP (PDB ID: 2I13)**. The individual ZF domains are shown in color and the DNA in space filled mode in dark blue and magenta. **(B)** TALEs (PDB: 3V6T). DNA strands are shown in dark blue and magenta. Each repeat is indicated with a different color. **(C)** CRISPR interacting with guide RNA (yellow) and target DNA (turquoise) (PDB: 4UN5). Some of the domains of the protein are indicated (topoisomerase domain in green and nuclease domain in purple). Residues interacting with the PAM sequence are shown in space filled mode.

Cys2–His2 ZFPs are made of modular zinc finger (ZF) domains where each finger domain is composed of one α-helix and two β-sheets coordinated by a zinc ion with two residues of cysteine and two residues of histidine. The α-helix of each finger domain is designed to recognize 3-bps of DNA. By exchanging the specific amino acid residues of the α-helix that make essential contacts with 3-bps of DNA ZFPs are then capable to bind a different DNA sequence. To recognize more specifically a target DNA sequence, finger domains can be linked together; for instance, a 6-ZFP protein (composed of 6 ZF domains) can recognize 18 bps of DNA, which mathematically represents a unique address in the genome (36) (Figures 1A and 2A).

One of the advantages of ZFPs is their modular architecture and small size (each ZFP motif is composed of 30 amino acid residues), which simplifies the production of the proteins and potentially their delivery into the cells (Table 1). In addition, the structure of artificial ZFPs is similar to the naturally occurring human ZFP transcription factors; therefore, their introduction into human cells is not anticipated to raise adverse immune responses. Our laboratory has demonstrated that engineered ZFPs are able to reactivate epigenetically silent genes such as class II tumor suppressor genes, which suggest that the ZFPs are able to reach the compact chromatin structure of silent genes (37–39). However, the up-regulation of *hyper*methylated tumor suppressor genes by artificial ZFPs (also commonly referred as artificial transcription factors, ATFs) is highly synergistic with combinations of epi-drugs (particularly the combination of decitabine, a DNMT3A inhibitor and vorinostat, a HDAC inhibitor), indicating that compact chromatin structure, indeed, represents a partial blockade for ZFPs (40, 41).

**Table 1:** **Comparison of DNA-binding backbones for genome engineering**.

ZFPs		TALEs		CRISPRs	
Advantages	Disadvantages	Advantages	Disadvantages	Advantages	Disadvantages
Small size: efficient delivery into the cells	Many off-targets	High specificity to the target	Sensitive to DNA methylation of the targeted region	Highly specific to the binding site and highly effective	
Cost and time effective synthesis		Successfully used in combination with the catalytic domain of many enzymes	Time consuming and more elaborate synthesis	DNA-binding domain is independent from effector domain	
Engineered from human proteins might be less susceptible to adverse immunoreactions			Engineered from bacterial backbones might elicit immunoresponses	Cost and time effective synthesis	Engineered from bacterial backbones might elicit immunoresponses
Successfully used in combination with catalytic domain of multiple enzymes			Big size might complicate delivery	Being used in combination with catalytic domain of any enzyme	Big size might complicate delivery
Successfully implemented in clinical trials			Susceptibility to DNA rearrangements		

The preference of ZFPs to their target sequences has been extensively studied since the early 2000s. For example, the ZFP targeting *ErbB2* gene from the Her-family specifically regulate the expression of *ErbB2* gene without altering the expression of other genes from the same family with similar sequences (*ErbB1*, *ErbB3*) (42). In addition, a study evaluating the effect of a ZFP targeting *checkpoint kinase 2* gene on the expression of 16,000 genes showed that the effect of the studied ZFP was specific to its target gene (43). The ErbB2-ZFP binding selectivity was analyzed using genome-wide ChIP-seq (chromatin immunoprecipitation sequencing). This study confirmed that the ZFP had the highest preference to the *ErbB2* gene among the other annotated genes (44). Despite the affinity of ZFPs to their preferred target genes, they might have off-targets too. In fact, the individual fingers of a ZFP might influence each other’s specificity (45), which brings up the need for more specific DNA-binding domains. In a recent study of engineered 6-ZFP proteins targeting the oncogene *SOX2* linked to the Krüppel associated box (KRAB) repressor domain, it was found that while the DNA-binding domain can potentially bind thousands of promoters in the cell, the ZFP was still capable of regulating a more limited subset of targets (46). This work suggested that the capacity of the ZFP proteins to regulate target genes was highly context-dependent. Hence, most of ZFP binding events were not associated with the target regulation. Interestingly, the ZFPs linked to the KRAB domain resulted in transcriptional repression of some genes, whereas for other loci binding was associated with target gene activation and with promoter demethylation. In sum, the physiological capacity of ZFPs to regulate target genes appear to be highly dependent on the chromatin context of the targeted region, including the presence of co-activator or co-repressors in that particular genomic address bound by the DNA-binding domain (46, 47).

In an effort toward the engineering of highly specific DNA-binding domains, transactivation like effector (TALEs), derived from the plant pathogen bacterial genus *Xanthomonas* were developed as novel modular DNA-binding proteins. TALEs act as transcription factors, which can bind to the promoters of disease resistance-related genes in plants, regulate their expression and cause infection in plant hosts (48). TALEs consist of a series of tandems repeats (33–35 amino acids), in which each repeat or module recognizes a single base pair (bp) of DNA (49) (Figures 1B and 2B). However, TALEs have their limitations including susceptibility to DNA rearrangements as consequence of their repetitive nature, and also their big size, which limits their delivery into the cells and target tissues (49, 50). In addition, it has been shown that a TALE targeting the *EGFP* gene fused to a DNA demethylase was not capable of demethylating the *KLF4* intron in a reporter plasmid (51). This might confirm the sensitivity of TALEs binding to methylated DNA. While one potential advantage of TALEs over the ZFPs is their higher structural complexity and their capacity to discriminate between closely related DNA sequences, one potential limitation is their lack of activity in certain genomic contexts (Table 1). In particular, TALEs are highly sensitive to DNA methylation, although re-engineering of the hypervariable DNA-binding regions might help to overcome this limitation (52).

In early 2013, another breakthrough technology, CRISPR–Cas (CRISPRs–CRISPR associated proteins), was introduced as a genome-engineering tool and has drawn great interest as an essential tool in molecular biology. The CRISPR–Cas system is a natural defensive molecular pathway in bacteria and archaea, which acts like an adaptive immune system against viral genome attacks. However, more than two decades of investigation was required to reasonably understand the molecular function of CRISPR–Cas. In 1987, it was first reported that *Escherichia coli* K12’s genome contains repetitive sequences adjacent to the alkaline phosphatase gene (53). In 2002, the CRISPR–Cas system was reported as the immune system of bacteria and archaea (54, 55). The CRISPR–Cas system can be classified into three types. The type II CRISPR–Cas9 is the simplest design and it is composed of one single endonuclease protein, Cas9, which is guided to a particular DNA sequence by small RNAs. This type II CRISPR–Cas9 (hereafter called CRISPRs) has been engineered and evolved as the framework for a new generation of DNA-binding domains (56). In essence, CRISPR is a RNA-dependent DNA-binding protein in which the information to bind the target gene is provided by a single synthetic 100-bp “guide” RNA. Guide RNAs recognize a target genomic sequence of approximately 20 bps upstream of a tri-nucleotide 5′-NGG-3′ protospacer adjacent motif (PAM) that is vital for recognition and specificity of guide RNAs. Guide RNAs form a complex with the endonuclease Cas9 protein, which induces DNA double-strand breaks. In fact, guide RNAs can direct the Cas9 protein to the target loci in the genome (Figures 1C and 2C). CRISPR technology is a flexible and cost-effective system for genome editing, which can be easily used to target multiple loci in a host genome. Importantly, CRISPRs seem to not be hampered by DNA methylation (57). In addition, it has been shown that the number of off-targets of CRISPRs in the human genome varies between 10 and <1000, and that the design of the guide RNA is essential in determining specificity (58). Interestingly, reducing the length of guide RNAs to 17–18 nucleotides increased the specificity of guide RNAs. These shorter guides showed similar or even higher efficacy than full-length guide RNAs (59). In order to increase the specificity of CRISPRs, dimeric RNA-guided *Fok*I nucleases (RFNs) were recently introduced. In this system, two split units of dCAs9-*Fok*I nuclease and two guide RNAs re-constitute a functional nuclease, and thus RFNs cleavage function is highly dependent on binding of two guide RNAs to the target DNA (60).

## Effector Domains for Genome Manipulation

For genome-editing purposes, DNA-binding domains such as ZFPs and TALEs are linked with nucleases (so called ZFNs and TALENs, respectively) and the resulting fusions are able to induce double-strand breaks in the targeted sequence in the genome. The breaks can eventually be exposed to the cellular repair system resulting in several insertions and deletions. The induced double-strand breaks using ZFNs and TALENs are associated with inheritable gene disruption, as it has been demonstrated in worms (61). Likewise, CRISPRs containing Cas9 nuclease facilitate double-strand cleavage at specific locations, which triggers the DNA repair program. Several genes of different organisms have been corrected or disrupted using ZFN, TALENs, and CRISPR technologies (50).

In order to modulate gene expression without altering the DNA sequence, both ZFPs and TALEs are exploited in the absence of a nuclease catalytically active domain. Instead, the proteinaceous DNA-binding domain is linked to an effector domain (ED), with either a transcriptional regulatory or epigenetic modifying effect. Similarly, to generate CRISPRs suitable for genome-engineering applications other than targeted double-strand DNA breaks, Cas9 is inactivated in its DNA cleavage domain by two mutations, D10A and H841A (62). The mutant or defective Cas9 (dCas9) with no endonuclease activity is directly linked to transcriptional or epigenetic modifying domains. We refer the CRISPRs system containing dCas9 as CRISPR–dCas9.

## Regulating the Expression of the Cancer Genome

By taking advantage of artificial DNA-binding technology, several cancer drivers have been targeted and transcriptionally modulated (Table 2). A wide range of EDs has been linked to ZFPs to up- or down-regulate the targeted genes. DNA-binding domains have been engineered with either transcriptional repressor domains (e.g., the Krueppel associated box (KRAB) domain) or transcription activator domains [e.g., the tetramer of herpes simplex virus protein VP16 (VP64)]. Transcriptional modulation of several tumor suppressor genes and oncogenes including *MASPIN*, *Her2/neu*, *SOX2*, *OCT4*, *EpCAM*, *ICAM-I*, and *C13Orf18* by ZFPs linked to the VP64 or KRAB domain have been reported in breast, ovarian, and lung cancer models. In these studies, down-regulation of overexpressed oncogenes and up-regulation of silent tumor suppressor genes using ZFPs fused to KRAB and VP64, respectively, resulted in reduced growth of cancer cells both *in vitro* and mouse models (39, 44, 47, 63–68). Likewise, TALEs and CRISPRs–dCas9 have demonstrated target gene modulation when linked to VP64 (51, 69) or KRAB (70, 71). Although each individual DNA-binding domain has been shown to be effective in up- or down-regulation, they act highly synergistically in combination. For example, the epigenetically silent *OCT4* gene has been very efficiently activated when different regions of its promoter are targeted by multiple TALE–VP64 molecules or by a CRISPRs–dCas9–VP64 in combination with several guide RNAs (72). Effective regulation has been achieved by targeting both core promoters and enhancer sequences (66).

**Table 2:** **Epigenome editing using catalytic domains of epigenetic enzymes fused to DBDs**.

DBD	Effector domain	Enzymatic function	Target gene/site	Epigenetic modification	Regulated expression	Reference
ZFP	DNMT3A	DNMT	*SOX2*	DNA methylation	Yes	(83)
ZFP	DNMT3A	DNMT	*EpCAM*	DNA methylation	Yes	(84)
ZFP	Dnmt3a-3L	DNMT	*VEGF-A*	DNA methylation	Yes	(104)
	Dnmt3a-C	DNMT	*VEGF-A*	Moderate methylation	Yes (moderate)	
TALEs	Tet1	DNA demethylase	*KLF4/RHOXF2*	DNA demethylation	Not assessed/yes	(51)
ZFP	Tet2	DNA demethylase	*ICAM-I*	DNA demethylation	Yes	(15)
TALEs	LSD1	Histone demethylase	*40 active enhancer*	Reduced of H3K4me2	On some	(86)
ZFPs	G9a	HMT	*Her2/neu*, *VEGF-A*	Increased H3K9me2	Yes	(44, 85)
ZFPs	Suvar39H1	HMT	*VEGF-A*	Increased H3K9me3	Yes	(85)
TALEs	Sirt3, NcoR	HDAC	*Neurog2*	Reduced H3K9ac	Yes	(69)
	PHF19	HMT-binding activity	*Neurog2*	Increased H3K27me3	Yes	(69)
	KYP	HMT	*Grm2*	Increased H3K9me1	Yes	(69)
	SID4X	Sin3 HDAC1 interaction domains	*Grm2*	Reduced H3K9ac	Yes	(69)
	TgSET8	HMT	*Grm2*	Increased H4K20me3	Yes	(69)
	NUE	HMT	*Grm2*	Increased H3K27me3	Yes	(69)
	HDAC8	Histone deacetylation	*Grm2*	Reduced H4K8ac	Yes	(69)
	RPD3	HDAC	*Grm2*	Reduced H4K8ac	Yes	(69)
	Sir2a	HDAC	*Grm2*	Reduced h4Kac	Yes	(69)
	Sin3a	HDAC1 interaction	*Grm2*	Reduced H3K9ac	Yes	(69)

*DBD, DNA-binding domain; DNMT, DNA methyltransferase; HMT, histone methyltransferase; HDAC, histone deacetylase; me, methylation; ac, acetylation*.

In contrast with ZFPs and TALEs, in which the DNA-binding domain is directly linked in frame with an ED, in the CRISPRs system the gene targeting activity is mediated by the guide RNA and the ED is linked to the dCas9 protein. Therefore, the guide RNAs and the dCas9-effector fusions are typically independently delivered into the cells. As a consequence, altering the targeted specificity of the CRISPRs does not require *de novo* protein engineering but just delivery of specific short guide RNAs. In addition, in order to enhance the efficiency of CRISPRs in regulating gene expression, several guide RNAs can be easily and quickly synthesized and combined with dCas9–ED fusions (73). The easy synthesis of guide RNAs facilitates construction of guide RNAs libraries for identifying the role of targeted genes in diseases or specific phenotypes. For example, a recent study used a library of 87,897 guide RNAs targeting 19,150 genes to introduce mutations using CRISPRs in the mice genome, which uncovered novel genes in the mouse genome modulating toxin susceptibility (74).

The relatively small number of off-targets of CRISPRs system makes it a unique research tool for genome manipulation. In addition, CRISPRs facilitates the simultaneous targeting of multiple loci, in a fast and economical manner for any laboratory today. Moreover, the choice of the particular class of DNA-binding domain is highly dependent on the ultimate research application and the nature of the targeted region in the genome. For example, a recent study compared the TALEs and CRISPRs–dCas9 targeting the enhancers of two pluripotency genes, *OCT4* and *NANOG*, for their efficiency in regulating the endogenous gene expression and in inducing cellular reprograming (75). Interestingly, TALEs were more efficient than CRISPRs–dCas9 in up-regulating these genes. Furthermore, CRISPRs–dCas9 was far less potent than TALEs targeting a similar genomic region in reprograming differentiated mouse embryonic fibroblasts into iPS (induced pluripotent) cells. In contrast, in the same study, CRISPRs–dCas9 were more efficient than TALEs in repressing the enhancer of these target genes. This study implies that the genomic region and the chromatin context are key factors in determining the binding efficiency of the artificial DNA-binding domains and also their effect in up- or down-regulation.

## Re-Wiring the Epigenome: A New Approach in Genome Engineering

The precise reversion of epigenetic modifications in a targeted and gene-specific manner (epigenome editing) has opened new and exciting avenues for cancer therapy. Indeed, the dynamic and reversible nature of epigenetic modifications offers the possibility to reprogram the pathology of the disease. Such epigenome reprograming can be potentially tailored to a specific subset of genes or patient groups. In addition, epigenome engineering allows for the modification or correction of gene expression patterns ideally in a durable and long-lasting manner, since some epigenetic modifications are mitotically transmitted from the mother cell to daughter cells. Lastly, targeting gene expression directly facilitates reactivation of tumor suppressor genes or the inhibition of elusive cancer drivers, for which no drug is currently available, such as transcription factors (e.g., *MYC*) and small GTPases (e.g., *RAS*).

The first epi-enzymes linked to artificial DNA-binding domains were the catalytic domains of DNA methyltransferases including DNMT3A and DNMT3B, which catalyze the *de novo* methylation of DNA, as well as prokaryotic DNA methyltransferases including M.SssI, M.*Hha*I, and M.*Hpa*II (76–82). More recently, 6-ZFP fusions linked to DNMT3A were shown to promote targeted methylation on *SOX2*, *MASPIN* (83), and *EpCAM* gene promoters (84). Interestingly, DNA methylation was associated with gene repression and in an oncogenic context resulted in cancer cell growth inhibition. In contrast, 6-ZFPs linked to DNMT3A targeting a tumor suppressor gene promoter resulted in enhanced tumor cell growth (83).

Recently, great attention has been placed on DNA demethylation mechanisms, including the characterization of many enzymes able to deaminate and remove the methylated cytosine (12, 14). The DNA demethylase Tet1 was engineered with TALEs targeting the *RHOXF2* gene, which led to the identification of the specific CpGs playing a role in gene expression (51). In another study, the DNA demethylase Tet2 fused to a ZFP was able to demethylate the *ICAM-1* gene (15), which was associated with gene up-regulation (Table 2). These studies indicate that epigenome editing can provide fundamental information on the role of specific epigenetic modifications in the control of gene expression in both normal and diseased cells.

Although DNA methylation plays an essential role in maintaining inactive chromatin, a complex language of histone post-transcriptional modifications re-enforce the effect of DNA methylation in gene silencing. The repressive histone modifications H3K9me2 and H3K9me3 were first targeted in the *VEGF-A* gene in HEK293 cells in 2002 (85) and more recently in the *Her2/neu* gene in breast cancer cells (44). In these studies, the catalytic domain of histone methyltransferase G9a or SUVAR-39-H1 were fused to 6-ZFP domains. Similarly, a more recent report demonstrated effective targeting of enhancers by lysine-specific demethylase 1 (LSD1) engineered with TALEs in order to identify the function of several enhancers and their chromatin state (86). Finally, a comprehensive set of 32 and 24 histone modifiers were fused to TALEs targeting the *Neurog2* and *Grm2* genes, respectively, to assess the role of the histone marks on regulation of gene expression [Ref. (69), Table 2]. These studies support the role of specific histone post-transcriptional modifications in gene expression regulation.

## Imaging of Human Loci Using CRISPRs

The human genome is dynamically and is spatially organized inside the nucleus and its spatio-temporal structure is critical in the regulation of gene expression. Heterochromatin and euchromatin positioning are obvious examples demonstrating that the higher-order of chromatin structure and nuclear organization underlie gene expression status. Fluorescently *in situ* DNA hybridization (FISH) is a powerful technique to image the location of a gene or fragment of the genome for genome analyses, although it is not applicable in living cells. In order to unravel the role of genome organization in gene expression, the DNA sequences in living cells can be chased and imaged using fluorescent DNA-binding domains. In 2013, the CRISPR–dCas9 system was successfully deployed to image repetitive elements in telomeres as well as in *MUCIN* genes (87). In this strategy, CRISPR–dCas9 is composed of guide RNAs binding specifically to the target loci and the dCas9 protein is fused to the green fluorescent protein (*GFP*) gene. By visualization of GFP, the expression of the CRISPR–dCas9 and its binding to the target locus in the genome was monitored in living cells. These applications of CRISPR technology open up new avenues for unraveling the mechanisms by which the higher order chromatin and the spatial organization of genome control gene expression.

## Potential of (Epi)Genome Engineering for Therapy and Disease: A Path to the Clinic

After extensive proof of concept of their efficiency in cell lines and animal models, ZFNs were qualified for clinical applications. For instance, ZFPs are being used for treatment of diabetic neuropathy and glioblastoma. ZFPs targeting HIV co-receptor CCR5 are in phase 1 clinical trials for the treatment of HIV/AIDS (88, 89).

In addition to ZFPs, TALENs are being used for introducing insertions/deletions in a targeted manner in animal, plant, and worm models (90), although their big size makes their delivery into the cells and tissues more problematic than ZFPs. Recently, CRISPRs have been shown to be highly efficient in gene correction in both human cell lines and animal models (91). For example, CRISPRs have been used to repair the metabolic enzyme gene *Fah* in hepatocytes, thereby correcting the disease phenotype in a mouse model (92). In another preclinical study, CRISPRs targeting and disrupting two genes (*Ppar-y* and *Rag1*) were injected into one-cell-stage monkey embryos. The engineered mutations in the two genes were confirmed in the genetically modified monkeys (93). Although CRISPRs have been shown to be excellent research tools for gene correction, to enter clinical trials issues such as human immune responses to the bacterial CRISPRs and targeted delivery should be carefully addressed. In this regard, there is an ongoing effort to alter some amino acid sequences of the Cas9 protein in order to decrease its size and decrease immune response in human cells.

A very recent preclinical study attempted the delivery of CRISPRs into mice liver. To deliver CRISPRs, a DNA vector encoding CRISPRs (the Cas9 and the guide RNA) were transferred into the blood via tail-vein injection, by which about 20% of hepatocytes are anticipated to receive the DNA. In this study, two tumor suppressor genes, *Pten* and *P53*, were targeted and mutated in the mice livers and, therefore, a cancer mouse model mimicking liver tumorigenesis was created. In the same study, the mice were injected with the CRISPRs targeting and correcting the mutant β*-catenin* gene, which is frequently mutated in liver cancer (94).

In another recent report, CRISPRs were able to target and destroy of Epstein–Barr virus (EBV) in patient-derived cells from a Burkitt’s lymphoma with EBV infection and the tumor cells showed reduced proliferation upon receiving the CRISPRs targeting EBV (95). In addition, CRISPRs could target and destroy human papillomavirus E6 or E7 oncogenes, which are integrated in the genome of cervical carcinoma cells. The E6 and E7 oncogenes induce the degradation of the tumor suppressor gene P53 and the destabilization of retinoblastoma protein (Rb), respectively, and cause cells to develop different types of cancer. The knockout of E6 and E7 by CRISPRs were associated with increased levels of P53 and Rb protein and increased cancer cell death (96, 97).

As mentioned above, CRISPRs’s natural function in bacteria and archaea is to destroy viral genomes. Similarly, CRISPRs have also been exploited to disrupt viral genomes, including the integrated HIV provirus (98, 99) and the hepatitis B virus (HBV) genome both *in vitro* and *in vivo* (100).

Clustered regularly interspaced short palindromic repeats were recently utilized to develop a rat model for Duchenne muscular dystrophy disease. Toward this aim, the *DMD* gene, which is located on the X chromosome and encodes dystrophin, was targeted and mutated using CRISPR–Cas9 (101). In this study, guide RNAs and Cas9 were co-injected into the zygote and the model carrying the intended mutation was developed. Importantly, CRISPRs were able to correct the mutant *DMD* gene in the germ line of a mouse model of Duchenne muscular dystrophy. This approach generated animals with 2–100% correction of the *DMD* gene, which was associated with a corrected phenotype (102). These models inducing directed mutations or corrected mutations in the targeted gene provide an excellent source of information for unraveling the mechanisms underlying disease progression.

The aforementioned preclinical and clinical studies suggest that targeted genome-editing tools are fast developing toward being translated into the clinic. One exciting possibility for such technology is the combination of (epi)genome engineering technology with current epigenetic (epi-drug) therapies. Epi-drugs are already approved for the treatment of patients with myelodysplastic syndrome, cutaneous T-cell lymphoma, and peripheral T-cell lymphoma. Recently, patients with solid tumors are also being recruited for treatment with epi-drugs and particularly with the combination of epigenetic inhibitors with hormone therapies or chemotherapy (23). However, an actual limitation of these treatments is the side effects of epi-drugs, which are, to some extent, due to their genome-wide effects. Thus, a goal of epigenome engineering technology is to improve the specificity of and potency of the epi-drugs while decreasing their dose and potential toxicity. For example, in *in vitro* studies, epigenetic enzymes or KRAB fused to the ZFP targeting *Her2/neu* gene showed synergistic effect with lapatanib in cell growth suppression of ovarian cancer cells (44).

Toward application of (epi)genome engineering technology in the clinic, one important challenge is the delivery of the chimeric DNA-binding proteins to target exactly the tumor in the patient’s body. To this aim, identifying the tumor subtype is a critical first step. Detecting tumor specific cellular receptors and investigating their mechanisms of action are of critical importance. Some types of receptors, like Her2/neu tyrosine kinase receptor, can internalize upon binding to its ligands. This ligand-mediated internalization is beneficial because it increases the level of drug administered to the tumor cells. This is, indeed, the major mechanism of function of trastuzumab, which can act as a ligand for this receptor in Her2/neu positive breast cancer (103). Similarly, the (epi)genome engineering tools could also be organized in a delivery package, e.g., via targeted nanoparticles coated with specific antibodies, which could be detected by receptors overexpressed on the surface of tumor cells of interest and thus, the unwanted effects of (epi)genome engineering tools in non-tumor cells could be reduced. Liposomes targeting sigma receptor overexpressed in ovarian cancer cells were able to effectively deliver an artificial-ZFP targeting a tumor metastasis suppressor gene (68). As nanotechnology research progresses, together with the development of quicker and cost-effective genomic sequencing, personalized treatments to tailor the cancer genome via genome-engineering approaches are becoming more than a fairytale but an exciting reality for cancer treatment.

## Conclusion

An aberrant landscape of epigenetic modifications is involved in cancer initiation and progression. Several epi-drugs are being used in clinical trials in order to reverse the disrupted epigenome of cancerous cells and reprogram the epi-pathology of the disease. However, epi-drugs have generalized genome-wide effects and therefore they can result in off-target effects and toxicity. In order to target epigenetic modifications at specific loci in the genome, the catalytic domain of epi-enzymes is linked to the sequence-specific DNA-binding domains by ZFPs, TALEs, and CRISPRs technology. Indeed, it is feasible today to generate DNA-binding domains to target virtually any sequence in the human genome. In addition to specific modulation of targeted loci, artificial DNA-binding domains also facilitate the discovery of novel genes involved in a phenotype or disease, and the imaging or detection of specific loci in the chromosomes. Precise epigenome editing has proved to be a successful research tool to ascertain the function of promoters and enhancers in gene regulation. However, the advent of genome sequencing has recently demonstrated that artificial DNA-binding domains may have substantial off-target binding activities. This limitation has rapidly forced the field to develop novel and highly specific DNA-binding domains. CRISPRs/dCas9 is the latest state of the art DNA-binding technology and it is associated with a small number of off-targets. In cancer, the modulation of gene expression by epigenome editing shows promising outcomes for the normalization of the phenotype of cancer cells. In addition, long-lasting targeting “non-druggable” oncogenes such as transcription factors is now possible by (epi)genome editing. In sum, the recent *in vitro*, *in vivo*, and clinical studies suggest that genome-engineering technology has begun to find its path toward the clinic.

## Conflict of Interest Statement

The authors declare that the research was conducted in the absence of any commercial or financial relationships that could be construed as a potential conflict of interest.

## Abbreviations

ac, acetylation; ChIP, chromatin immunoprecipitation; ChIP-Seq, ChIP sequencing; epi-drugs, epigenetic drugs; epi-enzymes, epigenetic modifying enzymes; me, methylation; P, phosphorylation; SUMO, SUMOylation; ubi, ubiquitination; ZFP, zinc finger protein.
